# HMGB1 Promotes Myeloid Egress and Limits Lymphatic Clearance of Malignant Pleural Effusions

**DOI:** 10.3389/fimmu.2020.02027

**Published:** 2020-09-04

**Authors:** Adam C. Soloff, Katherine E. Jones, Amy A. Powers, Pranav Murthy, Yue Wang, Kira L. Russell, Miranda Byrne-Steele, Amanda W. Lund, Jian-Min Yuan, Sara E. Monaco, Jian Han, Rajeev Dhupar, Michael T. Lotze

**Affiliations:** ^1^Department of Cardiothoracic Surgery, University of Pittsburgh School of Medicine, Pittsburgh, PA, United States; ^2^Cancer Immunology and Immunotherapy Program, UPMC Hillman Cancer Center, Pittsburgh, PA, United States; ^3^Department of Surgery, Division of Surgical Oncology, University of Pittsburgh School of Medicine, Pittsburgh, PA, United States; ^4^Departments of Immunology and Bioengineering, University of Pittsburgh School of Medicine, Pittsburgh, PA, United States; ^5^iRepertoire, Inc., Huntsville, AL, United States; ^6^Department of Cell, Developmental and Cancer Biology, Oregon Health and Science University, Portland, OR, United States; ^7^Division of Cancer Control and Population Sciences, UPMC Hillman Cancer Center, Pittsburgh, PA, United States; ^8^Department of Epidemiology, Graduate School of Public Health, University of Pittsburgh, Pittsburgh, PA, United States; ^9^Department of Pathology, University of Pittsburgh School of Medicine, Pittsburgh, PA, United States; ^10^Surgical Services Division, VA Pittsburgh Healthcare System, Pittsburgh, PA, United States

**Keywords:** HMGB1, malignant pleural effusions, benign pleural effusions, immune repertoire, tumor immunology, γδ T cells, adaptome, monocytes

## Abstract

Pleural effusions, when benign, are attributed to cardiac events and suffusion of fluid within the pleural space. When malignant, lymphatic obstruction by tumor and failure to absorb constitutively produced fluid is the predominant formulation. The prevailing view has been challenged recently, namely that the lymphatics are only passive vessels, carrying antigenic fluid to secondary lymphoid sites. Rather, lymphatic vessels can be a selective barrier, efficiently coordinating egress of immune cells and factors within tissues, limiting tumor spread and immune pathology. An alternative explanation, offered here, is that damage associated molecular pattern molecules, released in excess, maintain a local milieu associated with recruitment and retention of immune cells associated with failed lymphatic clearance and functional lymphatic obstruction. We found that levels of high mobility group box 1 (HMGB1) were equally elevated in both benign and malignant pleural effusions (MPEs) and that limited diversity of T cell receptor expressing gamma and delta chain were inversely associated with these levels in MPEs. Acellular fluid from MPEs enhanced γδ T cell proliferation *in vitro*, while inhibiting cytokine production from γδ T cells and monocytes as well as restricting monocyte chemotaxis. Novel therapeutic strategies, targeting HMGB1 and its neutralization in such effusions as well as direct delivery of immune cells into the pleural space to reconstitute normal physiology should be considered.

## Introduction

At advanced stages, many types of cancer can infiltrate the pleural cavity, disrupting the normal mechanism of fluid secretion and absorption, resulting in an unopposed collection of cancer containing fluid termed malignant pleural effusion (MPE). MPEs pose a major detriment to the quality of life for a cancer patient, are marked by persistent inflammation, associated with reduced life expectancy, and often heralding the terminal stages of cancer (1–4). While we are able to palliate some of the symptoms from MPEs, systemic treatments most often fail to reverse course and localized strategies are not often successful. The inflammatory milieu of MPEs may provide insight regarding tumor biology related to immune evasion and immune dysfunction (5). MPEs possess an abundance of immune cells, often exceeding 10^8^ leukocytes per effusion, that may harbor dormant effector cells which could be expanded and utilized for therapy. Unfortunately, our understanding of the phenotypes of immune cells, their tumor specificity, and how they are impacted by the MPE environment are only beginning to be understood. The lymphatics themselves, regulating pleural fluid dynamics, are not just passive conduits. Evolutionarily they emerged after the appearance of cartilaginous fish, with organized lymph nodes found later, primarily in mammals and in some birds. Pleural fluid is responsible for lubrication between the visceral and parietal layers. Water, and associated solute less than 4 nm, pass freely through mesothelial cells. Materials >1000 nm, are phagocytosed. Pleural lymphatics cycle pleural fluid at a rate of 0.4 mL/kg/h (6–10). In the pleural space of sheep, where it has been measured, effusions can be completely removed by the lymphatics in a linear manner at a rate of 0.28 mL/kg/h, ∼28 times greater than the rate of pleural fluid formation.

High mobility group box 1 (HMGB1) is a multifaceted nuclear protein that has diverse biological roles, regulating inflammation and orchestrating cellular immune responses (11–13). Outside the cell, HMGB1 functions as a prototypic damage-associated molecular pattern (DAMP) molecule with established roles in pathobiology such as cancer initiation and progression, toxic shock, and trauma (11–13). In the setting of cancer, HMGB1 is released from lysed and stressed cells into the extracellular space, causing chronic inflammation, attracting immune cells to the tumor site and engaging RAGE and Toll-like receptors to initiate and propagate inflammatory responses (12). HMGB1 can also be released by activated immune cells, which possibly contribute to the local pathophysiology. In a MPE, it is unknown whether HMGB1 has a role in anti-tumor immune activation or tumor progression (14). It may indeed serve as a vehicle to regulate lymphatic egress, limiting pathology to sites of tissue damage and preventing propagation of tumor and microbes.

During tumor development and therapy, pleiotropic HMGB1 mediates diverse biologic functions, promoting both cell survival and death by regulating unique signaling pathways (14). HMGB1 provides a protective role in cancer immunity by initially inducing immunogenic tumor cell death, contributing to immune-mediated eradication of tumors during their early development (15–17). Release of HMGB1 into the extracellular space contributes to the maturation of dendritic cells (DCs) and prompts cytotoxic T lymphocyte responses (18–20). In contrast, HMGB1 also plays an adverse role in tumor immunity. HMGB1 recruits and sustains immunosuppressive myeloid-derived suppressor cell and regulatory T cell populations during chronic inflammation. Sustained HMGB1 signaling limits chemotherapeutic responses in tumor cells, promoting resistance via enhanced autophagy, inhibiting both intrinsic and extrinsic-mediated apoptotic pathways in cancer cells (18–21). The presence of heightened levels of HMGB1 in human tumor tissues and in the circulation is frequently associated with disease severity and progression.

Inflammatory signaling among tumor cells, vasculature, and immune cells contributes to the development of pleural effusions (22). The pleural space is a sterile, secluded location in the thoracic cavity that is a frequent metastatic site for various histologic subtypes (2). The development of a MPE is the product of three associated processes; inflammation, lymphangiogenesis, and vascular leakage. More than 80% of MPEs feature elevated lymphocyte populations that play an important role in MPE pathogenesis (2, 3, 22). Lymphatic vessels respond to tumor and pathogen-induced changes in fluid transport, helping to regulate host immunity (23, 24). Based on these studies in melanoma and viral infection, we therefore hypothesized that, in the context of persistent tumor and other inflammatory mediators within the pleural space, that released DAMPs serve as cues to influence regional lymphatic vessel function, downstream immune induction, and host antitumor defense. Given the established role of HMGB1 and HMGB1-induced inflammation in the pathology of malignant disease, and the unique interface between immunity and tumor cells within the microenvironment established within MPEs, we examined the potential influence of HMGB1 on immune composition of MPEs.

## Materials and Methods

### Collection of Specimens

Informed consent for participation was obtained prior to effusion drainage from all patients, and no subjects were under the age of 18. The use of human tissue samples and the experiments were approved by the Institutional Review Board at the University of Pittsburgh (IRB#PRO16110093). Samples were collected as excess pathologic specimens and experiments were not performed on humans. Effusions were collected for clinically indicated drainage of symptomatic effusions, either by thoracentesis, or from a temporary or indwelling tunneled catheter. These specimens would otherwise be medical waste. All methods were carried out in accordance with relevant guidelines and regulations. Seventy pleural effusions resulting from malignant disease (*N* = 46) or benign etiology (*N* = 24) were included. Ten patients (*N* = 7 malignant and 3 benign), underwent repeated collection of samples between 6 and 301 days apart. Quantities of 350–1000 cc were processed immediately upon collection wherein red blood cells were lysed, and cell pellets and acellular fluid were isolated and preserved. All effusions were examined by a cytopathologist. For normal serum controls, subjects (*N* = 404) were drawn from two population-based cohorts, the Shanghai Cohort Study and the Singapore Chinese Health Study (25). Serum from a cohort of patients with metastatic clear cell renal cell carcinoma (ccRCC) (*N* = 30) were obtained in the context of an IRB approved protocol, 11-080 conducted within the Cytokine Working Group. These three cohort studies have been approved by the Institutional Review Boards of the Shanghai Cancer Institute, the National University of Singapore, and the University of Pittsburgh.

### HMGB1 ELISA

High mobility group box 1 levels in the acellular fractions of pleural effusions and sera were measured using a specific ELISA according to the manufacturer’s protocol (IBL International-Shino Test Corporation, Kandajimbocho Chiyoda-ku, Japan). All measurements were performed in duplicate.

### Cell Isolation and Culture

Peripheral blood mononuclear cells (PBMC) were isolated from consenting healthy volunteers using lymphocyte separation media (Corning). Cryopreserved PBMC were thawed, and γδ T cells were negatively selected (STEMCELL Technologies). γδ T cells were cultured for 10 days in complete media containing RPMI-1640, 5% human AB serum (GemCell), 1% Pen-Strep, and recombinant cytokines IL-2 (3000 IU/ml, aldesleukin, Clinigan), IL-15 (70 ng/ml, Miltenyi Biotech), and IL-21 (30 ng/ml, Miltenyi Biotech). γδ T cell phenotype was confirmed by flow cytometry and viable cultures with >80% γδ TCR^+^, <0.5% αβ TCR^+^, and <5% CD3^–^ CD56^+^ were utilized for subsequent studies. CD14^+^ monocytes were isolated via MojoSort positive magnetic bead isolation kit (BioLegend) per manufacturers protocol and cultured as described below in complete RPMI-1640 without additional cytokines. Acellular MPE fluid used for *in vitro* assays was generated by pooling three individual donors with the final solution containing 54.38 ng/ml HMGB1 as determined by ELISA. When used at 50% in our assays (1:1 with media), this yielded a final concentration of 27.19 ng/ml HMGB1 replicating median levels identified in our cohort.

### γδ T Cell Expansion

For expansion studies, 2 × 10^5^ γδ T cells were seeded in 24-well plates and cultured in complete media containing IL-2, IL-15, and IL-21 with or without addition of rHMGB1 (200 ng/ml, R&D Systems), acellular MPE fluid (50%), neutralizing anti-HMGB1 polyclonal chicken Ab (10 μg/ml, Tecan), or humanized CD3/CD28 agonist (20 μl/1:100, T cell TransActTM Miltenyi Biotech) as indicated. γδ T cells were cultured for up to 11 days incubated at 37°C, 5% CO_2_ and maintained at 0.5–1.5 × 10^6^ cells/well with rHMGB1, acellular MPE fluid, and anti-HMGB1 Ab added upon well splitting to maintain initial culture conditions. Live cell counts were determined with acridine orange and propidium iodide staining on day 6 and day 11 with an automated cell counter (Cellometer K2, Nexcelom Biosciences).

### Cytokine Analysis

To determine cytokine production, 2 × 10^5^ γδ T cells were washed in PBS and plated in 200 μl in a 96-well plate and cultured in cytokine free complete media with addition of rHMGB1, pooled acellular MPE fluid, anti-HMGB1 antibody, or CD3/CD28 agonist as indicated. Following 24-h incubation, 50 μl culture supernatant was assayed with the Th1/Th2/Th17 cytometric bead array kit (BD Biosciences) measuring IL-2, IL-4, IL-6, IL-10, TNFα, IFNγ, and IL-17A per the manufacturer’s instructions. Similarly, 5 × 10^4^ CD14^+^ monocytes were cultured in 100 μl in a 96-well plate using complete media in the presence or absence of acellular MPE fluid, rHMGB1, anti-HMGB1 Ab, or LPS (10 μg) (BioLegend) for 4-h. Subsequently, 50 μl culture supernatant was assayed using the human inflammation cytometric bead array kit (BD Biosciences) measuring IL-1β, IL-6, IL-8, IL-10, IL-12p70, and TNFα per the manufacturer’s instructions. Data was collected on a 5-laser Aurora (Cytek Biosciences) or 4-laser BD LSR Fortessa flow cytometers and analyzed with FlowJo V10.7 (BD Biosciences).

### Monocyte Migration

Chemotaxis of CD14^+^ monocytes was performed using a 96-well ChemoTX system (Neuroprobe) with membranes containing 8 μm pores per manufacturer’s protocol. Briefly, monocytes were rested overnight in serum-free RPMI-1640 then 10^5^ cells in 80 μl were plated onto top chamber of the membrane. Lower chambers contained 325 μl of complete RPMI-1640 with or without addition of rHMGB1 (200 ng/ml), anti-HMGB1 Ab (10 μg/ml), LPS (10 μg/ml), and pooled acellular MPE fluid (50%). ChemoTX plates were incubated at 37°C, 5% CO_2_, for 12 h. Transwell membranes were wiped with cotton and washed in PBS to remove unbound cells, then stained with 0.2% crystal violet. Transwell membranes were imaged using a Leica DMI 3000B digital microscope and cell quantitation performed by ImageJ (United States National Institutes of Health) to extrapolate cell counts of three randomly selected fields to represent the total area of the membrane well.

### Flow Cytometry

Immunophenotyping of pleural effusions was performed following cryopreservation of samples. All reagents were purchased from BioLegend unless otherwise specified. 1–5 × 10^6^ cells per sample were stained in Cell Staining Buffer using combinations of mAbs specific followed by labeling with amine-reactive viability dye (LiveDead, Molecular Probes). To determine leukocyte composition in pleural effusions, cells were labeled with mAbs specific for: EpCAM (9C4), CD45 (HI30; BD Biosciences), CD3 (UCHT1), CD4 (RPA-T4; Invitrogen), CD8a (RPA-R8), HLA-DR (L243), CD11b (ICRF-44), CD14 (HCD-14), CD16 (3G8), CD15 (W6D3), CD66b (G10F5; BD Biosciences), CD123 (6H6), CD11c (3.9), CD56 (HCD56; BD Biosciences), and CD19 (SJ25C1). Lineage gating for DCs includes CD3, CD19, and CD56. Samples were fixed in 1% paraformaldehyde and data was collected on a five laser LSR Fortessa (BD Bioscience). FlowJo (BD) software was used for conventionally gated data analysis.

### TCR Repertoire Analysis

The cellular component of MPEs was isolated, cryopreserved, and transported to iRepertoire for T cell receptor (TCR) analysis. RNA extraction from MPE cells using a RNeasy Micro Kit (Qiagen, Valencia, CA, United States) according to the manufacturer’s instruction. RNA concentrations were measured by spectrophotometry. iRepertoire multiplex primer sets (iRepertoire, Inc. Huntsville, AL, United States) were used to amplify the CDR3 region of TCR α, β, γ, and δ chains by using RNA as template as described by Wang et al. (26). The whole amplification process and library preparation process for next generation sequencing were fully automated in the iR-Procecessor and iR-Cassette (iRepertoire, Inc.). Then, paired-end sequencing was performed on purified PCR products using an Illumina MiSeq v2 300-cycle Reagent Kit (Illumina Inc.), for an average read depth of 30,000 reads per sample. Raw cDNA sequences were first analyzed to identify V and J genes by using iR-map and visualized in iRweb (iRepertoire, Inc.). Multiple alignments and hierarchical clustering of conserved amino acid sequences were analyzed as described (26). RNA samples were split into two reactions and processed as technical replicates. The coefficient of determination (*R*^2^) was calculated by linear regression to show the correlation between the replicates of CDR3 frequencies prior to data analysis to exclude PCR and sequence errors. The diversity of the TCR repertoire was calculated based on the diversity index (DI) defined mathematically by Wu et al. (27). Tree maps were used to reveal the diversity and characteristics of TCR repertoire. In a tree map, each rounded rectangle represents a unique V–J combination of uCDR3, where the size of a spot denotes the relative frequency.

### Statistical Analysis

All results were expressed as means ± standard error of the mean (SEM) unless otherwise stated. Data were analyzed using non-parametric Mann–Whitney U tests for comparisons of patient groups, paired Student’s *t*-test for analysis of change in HMGB1 levels and cell densities within patients over time, unpaired Students *t*-test for analysis of healthy donor cell expansion, cytokine production, or migration *in vitro*, or Spearman rank-order correlation tests performed using GraphPad Prism8 (GraphPad Software). For all hypothesis tests, a *p* < 0.05 was considered statistically significant.

## Results

### HMGB1 Levels Are Elevated in Pleural Effusions

**FIGURE 1:**
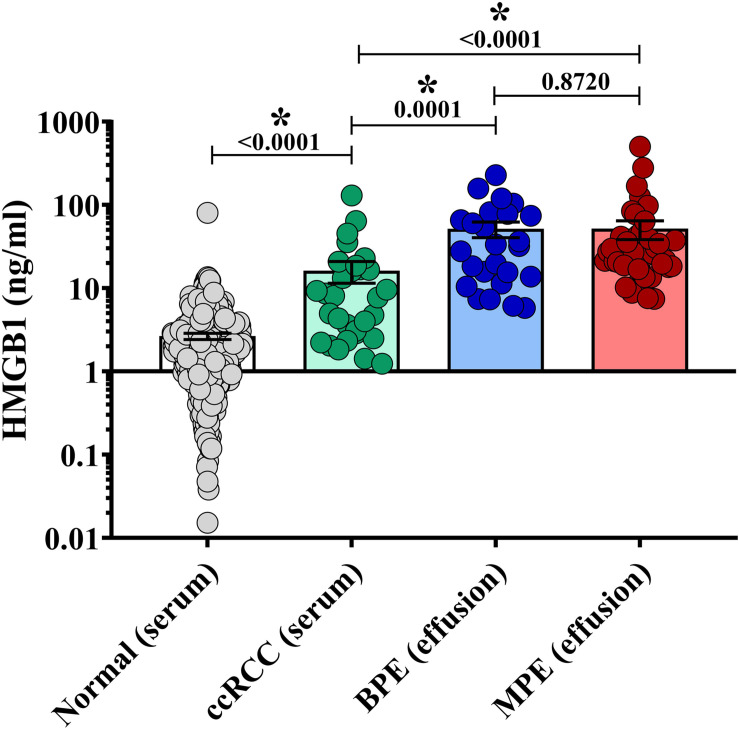
HMGB1 levels are elevated in pleural effusions. HMGB1 levels were measured in pleural effusions from patients with metastatic malignant disease (MPE) (*N* = 46), in effusions from patients presenting with congenital heart failure or effusion of benign origin (BPE) (*N* = 24), within the sera of healthy donors (*N* = 404) and sera from patients with metastatic clear cell renal cell carcinoma (ccRCC) (*N* = 30) by specific ELISA. Levels of HMGB1 in the sera of ccRCC patients (16.16 ng/ml ± 4.708) were significantly higher than those of healthy controls (2.64 ng/ml ± 0.229) (*p* = 0.001) HMGB1 was comparable between pleural effusions (*p* = 0.872), BPE containing 53.07 ng/ml ± 11.21 and MPE possessing 48.89 ng/ml ± 12.31. HMGB1 levels in serum from ccRCC patients, BPE, and MPE were all significantly elevated compared to healthy controls (*p* ≤ 0.0001). Data represent means ± SEM and Mann–Whitney U tests were used for comparisons. *denotes statistical significance.

To characterize levels of soluble HMGB1 in pleural effusions from malignant and benign etiologies, an HMGB1-specific ELISA was performed on the acellular fraction of pleural effusions from patients with metastatic MPE (*N* = 46) or benign pleural effusion (BPE) (*N* = 24). MPEs were secondary to various primary cancers including breast (*N* = 19), lung (*N* = 11), ovarian (*N* = 8), sarcoma (*N* = 3), salivary gland (*N* = 1), and colon (*N* = 1) (Table 1). As a reference control, we measured HMGB1 levels in the serum of healthy donors (*N* = 404) and patients with metastatic ccRCC (*N* = 30). Levels of HMGB1 in the sera of ccRCC patients (16.16 ng/ml ± 4.708) were significantly higher than those of healthy controls (2.64 ng/ml ± 0.229) (*p* ≤ 0.0001) (Figure 1). Notably, levels of HMGB1 in pleural effusions were significantly higher than serum HMGB1 in both healthy and ccRCC cohorts (*p* = ≦0.0011), irrespective of effusion etiology (Figure 1). Soluble HMGB1 was comparable between pleural effusions (*p* = 0.872), with BPEs containing 53.07 ng/ml ± 11.21 and MPEs containing 48.89 ng/ml ± 12.13, respectively (Figure 1). HMGB1 levels in sera from ccRCC patients, BPEs, and MPEs were all significantly greater than those detected in healthy control sera (*p* ≤ 0.0001). Additionally, in our cohort, intrapleural HMGB1 from lung cancer patients (70.05 ng/ml ± 24.70) was significantly elevated compared to levels observed in ovarian cancer patients (21.01 ng/ml ± 3.54; *p* = 0.025) and raised compared to breast cancer patients (34.50 ng/ml ± 7.38; *p* = 0.057) (Supplementary Figure 1). No differences were observed between HMGB1 levels in MPEs secondary to breast or ovarian cancers.

**TABLE 1:** Characteristics of patients with pleural effusion.

	Number of Patients	MPE	BPE	*p*-Value (MPE/BPE)
Total number of patients	70	46 (65.7)	24 (34.2)	
Age	65.89 (±1.46)	64.6 (±2.03)	68.3 (±1.70)	0.176
**Gender**				
Male	21 (30.0)	6 (13.0)	15 (62.5)	
Female	42 (70.0)	40 (87.0)	9 (37.5)	
**HMGB1**				
Mean ± SEM	50.33 (±8.90)	48.89 (±12.31)	53.07 (±11.21)	0.872
Median	26.25	22.88	32.78	
**Effusion cells**				
Tumor (% total)	25.74 (±6.07)	33.10 (±7.03)	0 (±0.0)	0.000
Leukocytes (% total)	63.70 (±5.96)	56.90 (±6.83)	87.50 (±5.59)	**0.007**
**Peripheral blood**				
Neutrophil (10^9^/L)	7.897 (±1.19)	6.778 (±1.48)	10.76 (±1.76)	**0.006**
Neutrophil (%)	73.61 (±1.93)	70.43 (±2.38)	81.73 (±2.38)	**0.002**
Lymphocyte (10^9^/L)	1.008 (±0.10)	1.065 (±0.13)	0.861 (±0.10)	0.861
Lymphocytes (%)	13.68 (±1.54)	15.42 (±2.08)	9.211 (±1.40)	0.123
Monocytes (10^9^/L)	0.674 (±0.05)	0.645 (±0.07)	0.748 (±0.06)	0.129
Monocytes (%)	8.934 (±0.83)	9.649 (±1.10)	7.117 (±0.78)	0.062
**Effusion etiology**				
Breast cancer		19 (41.3)		
Lung cancer		11 (23.9)		
Ovarian cancer		8 (17.4)		
Sarcoma		3 (6.52)		
Colon cancer		1 (2.17)		
Salivary gland cancer		1 (2.17)		

*Values are represented as *n* (%) and mean(±SEM) unless noted. *p*-values are derived using a Mann–Whitney U test. Bold values are statistically significant.*

### Association of Intrapleural HMGB1 and Cell Density of Pleural Effusions

**FIGURE 2:**
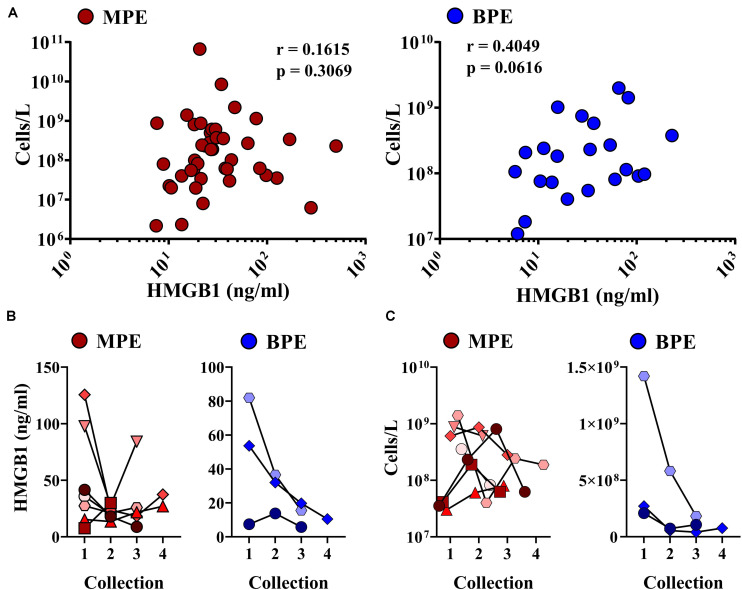
Correlations between intra-pleural HMGB1 and gross cellularity of pleural effusions. **(A)** Levels of intra-pleural HMGB1 did not predict cellularity as cells/liter of MPEs (left, red), but were significantly correlated with cell density in BPEs (right, blue). **(B)** When measured serially following multiple drainage events of pleural effusions, HMGB1 decreased in BPE (right, blue). No consistent trend was observed in HMGB1 levels longitudinally in MPE (left, red). **(C)** Over time, cell density decreased in both MPE (left, red) and BPE (right, blue) when serially sampled. Correlations were performed using Spearman rank-order tests.

We next examined associations between HMGB1 levels detected in MPEs and BPEs and gross cellularity of the effusion. As total cell number and volume of each effusion varies dramatically, values were represented as total live cells per liter of effusion. Although there was no association between HMGB1 and cell density within MPEs, a minor correlation between BPE cell density and HMGB1 was identified within our cohort (*p* = 0.061) (Figure 2A). To examine the temporal dynamics of this interaction, serial effusions were collected up to four times from patients with MPE (*N* = 7) or BPE (*N* = 3). HMGB1 levels and intrapleural cell densities in MPEs were highly variable over time, whereas we observed a continual decrease in both HMGB1 levels and gross cellularity in BPEs upon serial drainage (Figures 2B,C). To evaluate if the state of the effusion microenvironment, i.e., HMGB1 levels, was associated with a systemic phenotype, neutrophil, lymphocyte, and monocyte levels were measured in the peripheral blood by complete blood count (Table 1). In patients with MPE, intrapleural HMGB1 positively correlated with increased lymphocytes, percentage (*p* = 0.019) and number (*p* = 0.005), and percentage of neutrophils (*p* = 0.021) in the circulation. By contrast, HMGB1 levels within BPEs were not associated with the percentage or numbers of these cellular subsets in the peripheral blood.

### HMGB1 Expression Is Associated With Unique Leukocyte Profile Within MPEs

**FIGURE 3:**
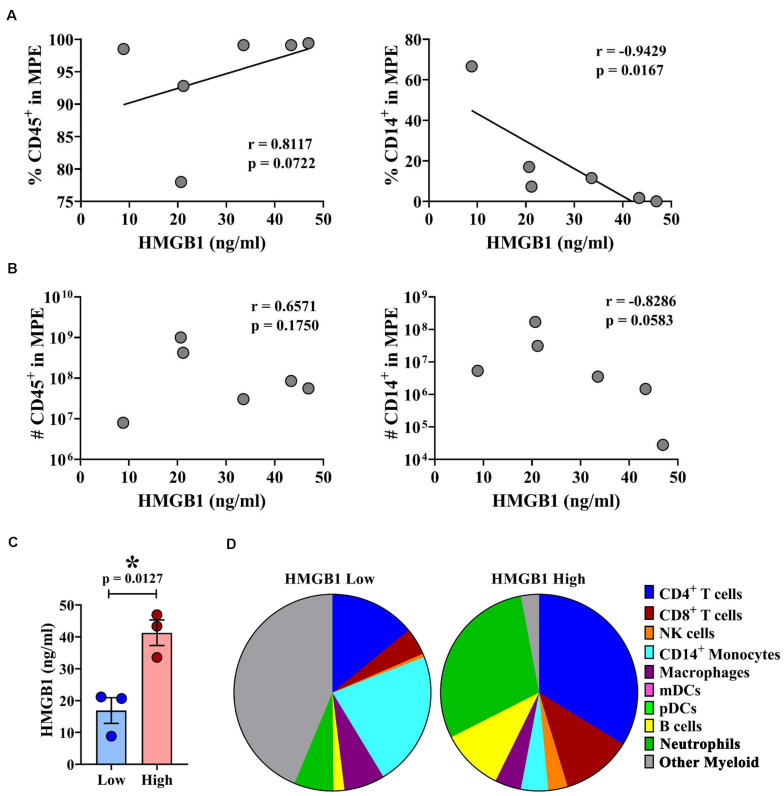
HMGB1 levels are associated with leukocyte infiltration and unique immune composition within MPEs. Multiparametric flow cytometry was used to immunophenotype cells isolated from MPEs. **(A)** The proportion and **(B)** absolute number of CD45+ leukocytes (left) was found to increase with greater levels of intra-pleural HMGB1. By contrast, **(A)** the proportion and **(B)** absolute numbers of CD14+ monocytes within MPEs declined with increased HMGB1. **(C)** Patients were segregated into HMGB1 low (16.88 ng/ml ± 4.03) and HMGB1 high (41.28 ng/ml ± 4.00) groups for comparative analysis. **(D)** Immune composition as proportion of CD45+ cells determined by select markers by flow cytometry of MPE-resident leukocytes in patients with low or high intra-pleural HMGB1 (*N* = 3 each), respectively. Group means were compared using a Mann–Whitney U test and correlations performed using Spearman rank-order tests. * denotes statistical significance.

Malignant pleural effusions contain a highly heterogeneous population of innate and adaptive immune cells, representing diverse states of activation. As HMGB1 is a potent inflammatory mediator capable of inducing chemotaxis, we performed comprehensive immunophenotyping on a subset of MPEs. Polychromatic flow cytometry was used to simultaneously identify CD4^+^ and CD8^+^ T cells (CD3^+^), NK cells (CD3^–^CD56^+^), monocytes (CD14^+^), macrophages (CD11b^+^CD66b^–^), myeloid DCs (Lin^–^HLA-DR^+^CD11c^+^), plasmacytoid DCs (Lin^–^HLA-DR^+^CD123^+^), B cells (CD19^+^), and neutrophils (CD66b^+^ CD16^+^). Correlative analysis was performed to identify associations between intrapleural HMGB1 and unique cell populations within the MPE. HMGB1 levels in MPEs were associated with increased presence, as both proportion of all cells as well as total numbers of CD45^+^ leukocytes within effusions (Figures 3A,B). Additionally, we observed a strong inverse correlation between the concentration of intrapleural HMGB1 and the proportion and absolute number of CD14^+^ monocytes (Figures 3A,B). When these subjects were stratified into HMGB1 high (41.28 ng/ml ± 4.03) and HMGB1 low (16.88 ng/ml ± 4.03) groups (*N* = 3 patients each) based on the median HMGB1 level in our MPE cohort (22.88 ng/ml) (Table 1), we observed pronounced differences in MPE immune composition (Figures 3C,D). Notably, patients with high levels of intrapleural HMGB1 were found to have greatly increased proportion of neutrophils (19.9% vs 5.4%), T cells (30.6% vs 15.5%), and B cells (6.8% vs 1.4%) compared to HMGB1 low subjects. By contrast, MPEs containing low HMGB1 were composed predominantly of monocytes (18.6% in low vs 3.0% in HMGB1 high) and a major population of undefined myeloid cells (36.6% vs 2.0% in HMGB1 high), likely to represent myeloid-derived suppressor cell populations.

### MPE Fluid Inhibits Monocyte Migration and Cytokine Production

**FIGURE 4:**
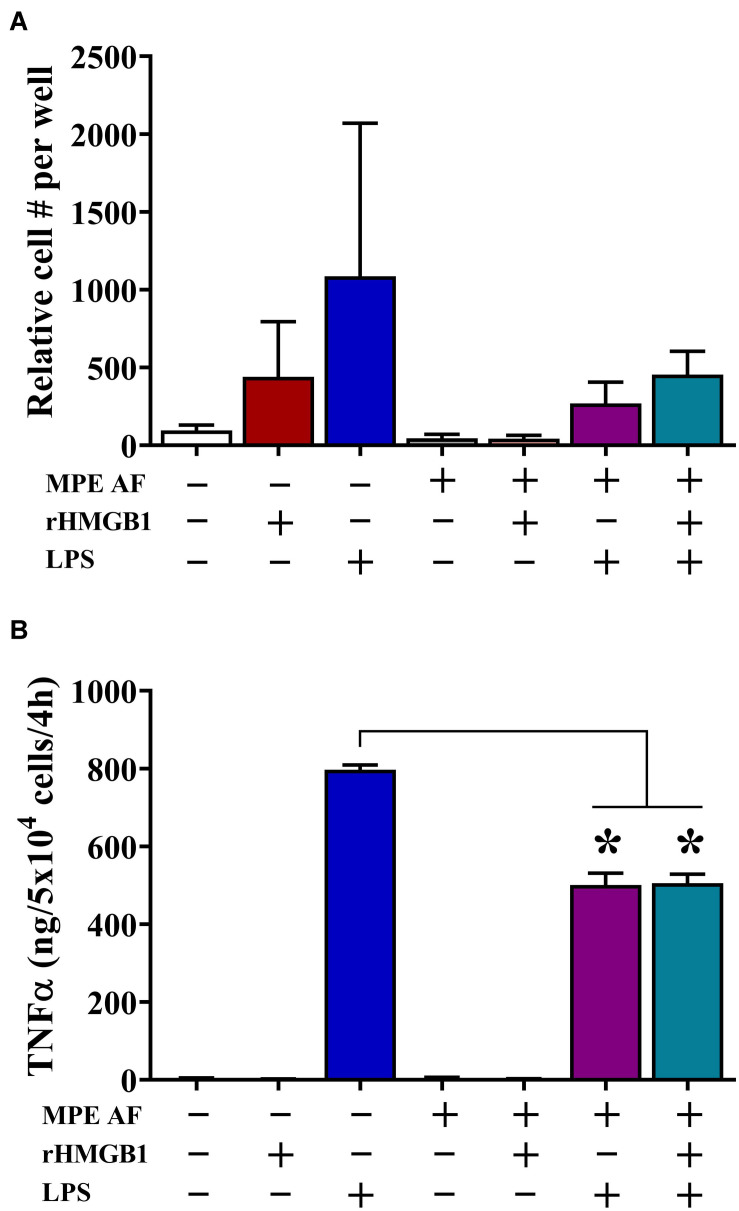
Acellular MPE Fluid Inhibits Monocyte Migration and TNFα Production Independent of HMGB1. The effect of HMGB1 and acellular MPE fluid (MPE AF) was assessed on healthy donor peripheral blood CD14^+^ monocyte function. **(A)** Twelve-hour monocyte migration (10^5^ cells/well) was evaluated in the presence of rHMGB1 (200 ng/ml), LPS (10 μg/ml), and 50% acellular MPE fluid (*N* = 5 repeats). While rHMGB1 and LPS enhanced migration, addition of acellular MPE fluid dramatically inhibited monocyte chemotaxis. **(B)** Soluble TNFα was measured via cytometric bead array following 4-h *in vitro* stimulation of 5 × 10^4^ monocytes (*N* = 3 repeats). In the presence of acellular MPE fluid, TNFα production following LPS stimulation (10 μg/ml) was significantly decreased irrespective of additional treatment with rHMGB1. *p* < 0.05. Data represent means ± SEM and Mann–Whitney U tests were used for comparisons. * denotes statistical significance.

We observed that intrapleural HMGB1 levels were associated with unique immune composition within MPEs, including decreased percentage and absolute numbers of monocytes. Consequently, we evaluated the effect of HMGB1 and acellular MPE fluid on chemotaxis and cytokine production from CD14^+^ monocytes isolated from the peripheral blood of healthy donors. Herein, monocyte migration was measured via ChemoTX Transwell membrane system following a 12 h culture in the presence or absence of 50% acellular MPE fluid, exogenous rHMGB1 (200 ng/ml), high dose LPS (10 μg/ml), or combinations of these factors (Figure 4A). LPS was evaluated as a known surrogate for other TLR4 ligands such as HMGB1 and taxanes and potent inducer of inflammatory responses in monocytes. In the absence of acellular MPE fluid, both rHMGB1 and LPS induced robust monocyte migration (Figure 4A). Notably, monocyte migration was dramatically inhibited in the presence of acellular MPE fluid with migration induced by rHMGB1 and LPS reduced by 90.3 and 60.1%, respectively (Figure 4A). Culture with rHMGB1 and LPS combined was insufficient to regain migratory ability (Figure 4A). As expected, blocking HMGB1 in MPE culture using HMGB1-specific antibody failed to enhance migration (data not shown). We next determined the capacity of monocytes to produce inflammatory TNFα following 4 h culture in the above conditions. Treatment of monocytes with LPS induced robust TNFα production measured in culture supernatants by cytometric bead array. Notably, addition of acellular MPE fluid reduced the levels of TNFα generated by monocytes following LPS stimulation by 37.1%, an effect that was independent of further addition of rHMGB1 (36.6% reduction) or anti-HMGB1 blocking antibody (43.6% reduction) (Figure 4B, and data not shown). Collectively, these findings suggest that the local microenvironment of MPEs exerts profound inhibitory effects upon monocyte functionality that are independent upon the presence of HMGB1.

### High HMGB1 Levels Are Associated With Reduced Diversity of γδ TCRs Within MPEs and γδ T Cell Proliferation *in vitro*

**FIGURE 5:**
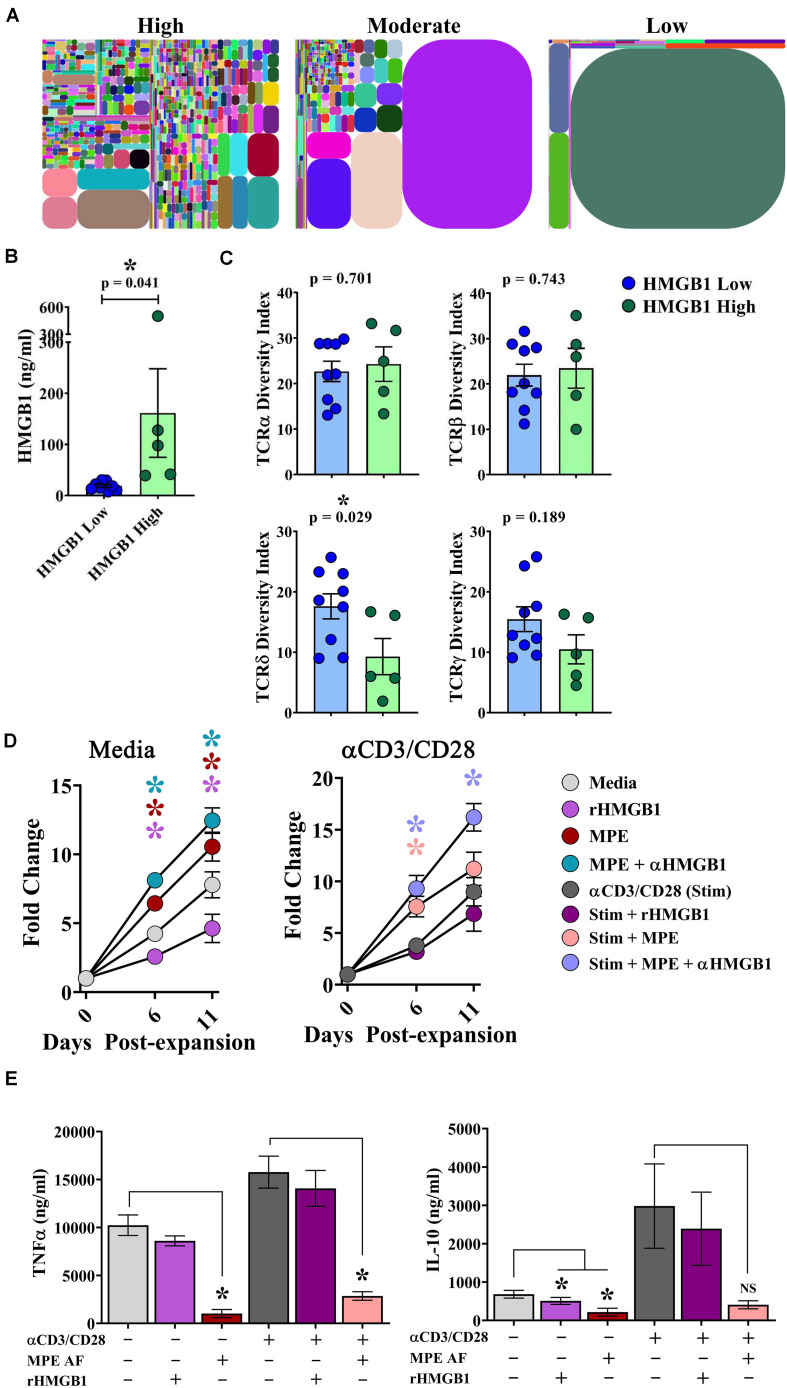
HMGB1 is associated with reduced γδ TCR diversity within MPEs and inhibition of γδ T cell proliferation *in vitro*. Amplification and next generation sequencing of the α, β, δ, and γ TCR chains was performed from bulk cells isolated from 14 MPEs. **(A)** Clonal diversity represented via tree maps illustrating the relative frequency of unique CDR3s as geometric shapes in patients with high, moderate, and low diversity of TCRδ chains within MPEs. **(B)** For comparative analysis, patients were segregated into HMGB1 low (*N* = 9) (18.75 ng/ml ± 2.68) and HMGB1 high (*N* = 5) (161.5 ng/ml ± 86.6) groups. **(C)** Diversity of TCRα and TCRβ chains, as calculated by diversity index, within cells of the MPE was not different between patients with high or low intrapleural HMGB1 (*p* = 0.743) (top). Diversity indices for TCRδ and TCRγ chains were significantly decreased in patients with high compared to low intrapleural HMGB1 (*p* = 0.029 and 0.189, respectively). **(D)** Expansion of cultured γδ T cells isolated from peripheral blood of healthy donors (*N* = 5) was observed over 11 days in the presence of rHMGB1 (200 ng/ml), anti-HMGB1 Ab (10 μg/ml), CD3/CD28 agonist (1:100 transact), and acellular MPE fluid (50%). rHMGB1 decreased unstimulated γδ T cell growth, while addition of acellular MPE fluid enhanced proliferation that was further increased with addition of anti-HMGB1 blocking antibody. Similar trends were observed in CD3/CD28 agonist stimulated γδ T cells. **(E)** Soluble TNFα and IL-10 in culture supernatants was determined by cytometric bead array following 24-h *in vitro* culture of 2 × 10^5^ γδ T cells under the above conditions. rHMGB1 had no effect on cytokine production; however, in unstimulated and stimulated γδ cells, acellular MPE fluid decreased both TNFα and IL-10 production. Cytokine production studies were completed in *n* = 3 different donors. Group means were compared using a Mann–Whitney U test with significance of *p* ≦ 0.05. * denotes statistical significance.

The clonal repertoire of T cells establishes diversity responding to pathogenic and endogenous insults. In the context of malignant disease, a greater breadth of TCR diversity may afford superior protection from tumor cells expressing neoantigens (28). To examine the effects of intrapleural HMGB1 on TCR diversity within pleural effusions, cells isolated from MPEs from 14 patients underwent multiplexed TCR amplification followed by next generation sequencing providing comprehensive detection of unique TCR clones. Between patients, we observed a wide range of TCR diversity, as illustrated in representative tree plots of the TCRδ chain from individuals with high, moderate, or low DIs, respectively (Figure 5A). Patients were then separated into HMGB1 high (*N* = 5; 161.5 ng/ml ± 86.6) and HMGB1 low (*N* = 9; 18.75 ng/ml ± 2.68) groups based on the MPE cohort median level of HMGB1 as before (Figure 5B). Heterogeneity of the TCR α and β chains was unaffected by levels of intrapleural HMGB1 in MPEs (Figure 5C). Interestingly, patients with high intrapleural HMGB1 levels had significantly diminished diversity indices for both TCRδ and γ chains present on T cells within MPEs (Figure 5C). Given that a decrease in TCR diversity may be associated with emergence of dominant clonotypes, we subsequently examined the 10 most highly expressed TCRδ clones from the five patients with high HMGB1 levels and corresponding low TCRδ diversity. Notably, the 10 dominant TCRδ clones per MPE were unique to each patient, with no individual clone being shared amongst individuals.

To examine the effect of HMGB1 on γδ T cell function, γδ T cells were isolated from the peripheral blood of healthy donors and assessed for their proliferative capacity and cytokine production in the presence or absence of rHMGB1 (200 ng/ml), anti-HMGB1 blocking antibody (10 μg/ml), acellular MPE fluid (50% of culture media), and polyclonal activation with a CD3/CD28 agonist. In media containing IL-2, IL-15, and IL-21, γδ T cells underwent a ∼7.5-fold expansion by 11 days in culture (Figure 5D). Notably, addition of rHMGB1 reduced γδ T cell expansion by ∼41% at day 11 (*p* = 0.005) compared to media control, whereas provision of acellular MPE fluid increased γδ T cell numbers by ∼35% (*p* = 0.005). Depletion of HMGB1 from acellular MPE fluid in culture further enhanced γδ T cell yield to ∼60% (*p* = 0.0005) by day 11. When γδ T cell culture was performed in the presence of an activating CD3/CD28 agonist, MPE fluid again augmented γδ T cell proliferation, with the highest γδ T cell numbers obtained following culture in HMGB1-depleted acellular MPE fluid with a 92% increase over CD3/CD28 agonist alone (*p* = 0.0006) (Figure 5D). Culture of activated γδ T cell with rHMGB1 decreased proliferation by 21% compared to CD3/CD28 agonist treated cells but did not attain statistical significance. Collectively, these findings suggest that HMGB1 inhibits γδ T cell growth which is conversely augmented by unidentified factors present within the acellular MPE environment.

We next asked whether HMGB1 present in culture media or acellular MPE fluid could effect cytokine production from γδ T cells. We determined cytokine levels by cytometric bead array in supernatants following 24 h culture of 2 × 10^5^ γδ T cells in the above conditions. Culture of γδ T cells in IL-2, IL-15, IL-21 containing media resulted in robust TNFα production that was further enhanced upon stimulation with CD3/CD28 agonist (Figure 5E). Notably, addition of acellular MPE fluid, with or without anti-HMGB1 antibody, significantly decreased TNFα production from both unstimulated and stimulated γδ T cells (*p* ≤ 0.002 for both conditions; data not shown) (Figure 5E). Provision of exogenous rHMGB1 did not effect TNFα production. Similarly, production of IL-10 by γδ T cells was enhanced by CD3/CD28 agonist stimulation, and again, substantially inhibited by the presence of acellular MPE fluid with or without anti-HMGB1 (*p* < 0.05) (Figure 5E). Culture with rHMGB1 did not effect IL-10 production by γδ T cells in this setting. Although provision of acellular MPE fluid profoundly inhibits cytokine production form γδ T cells, this effect seems to be independent of HMGB1.

## Discussion

Various pathologic processes regulate the development of pleural effusions. To assess the presence of the prototypic DAMP, associated with high levels of unscheduled cell death and/or cellular stress, within the unique tumor microenvironment of MPEs, we quantified intrapleural HMGB1 using BPEs as a comparator. Previous studies have shown that transudative effusions resulting from congestive heart failure or liver cirrhosis had significantly lower levels of HMGB1 when compared with exudative effusions arising from infection or malignancy (29, 30). Although levels of intrapleural HMGB1 varied between studies (15.0–36.62 ng/ml transudate), (35.1–118.0 ng/ml infectious), and (29.6–111.45 ng/ml malignancy), quantities were similar to those identified in our local cohort with expected variability due to limited sample sizes (29, 30). We found, compared to reference cohorts from sera of both healthy controls and metastatic ccRCC patients, that intrapleural HMGB1 levels in both MPEs and BPEs were significantly elevated. Notably, intrapleural HMGB1 was comparable in effusions resulting from malignant and benign processes. Because both malignant and benign effusions can have a component of associated inflammation, it is of interest that MPEs and BPEs demonstrate increased levels of HMGB1, whereas HMGB1 is rapidly cleared from serum in the setting of trauma. As such, the presence of elevated HMGB1 may serve as a novel therapeutic target if further studies implicate DAMPs in the pathologic subversion of fluid reabsorption in the pleural cavity.

High mobility group box 1 has both immune stimulating and suppressing properties (31). HMGB1 promotes maturation and subsequent cell death in macrophage−derived DCs (32). HMGB1 also enhances the function of regulatory T cells via enhanced IL-10 production, while inhibiting the effector function of conventional T cells including IFNγ production and proliferation (21, 33). Activated macrophages, natural killer cells, and mature DCs can release HMGB1, which may promote a positive feedback loop to propagate subclinical inflammation, tumor initiation and progression (34–38). Collectively, the immunosuppressive effects of HMGB1 serve to inhibit the generation of *de novo* tumor-specific immunity, as well as suppress the maintenance of pre-existing anti-tumor responses. In MPEs, HMGB1 levels are associated with an increase in leukocyte infiltration with reduced monocyte numbers. Notably, the presence of acellular MPE fluid alone restricted monocyte chemotaxis and reduced inflammatory cytokine release *in vitro*, and may, in the setting of chronic HMGB1 exposure *in vivo*, induce apoptosis in effusion-resident myeloid cells. In our cohort, high intrapleural HMGB1 was associated with increased T cell, B cell, and neutrophil recruitment into the MPEs, mirrored by increased number of lymphocytes and neutrophils in the systemic circulation, suggesting that the chemotactic properties of HMGB1 propagated an active inflammatory environment with substantial involvement of adaptive immune cells. In contrast, low intrapleural HMGB1 was associated with a significant proportion of undefined myeloid cells, likely representing various myeloid-derived suppressor cell populations. Future mechanistic studies will be required to define the causal effect of intrapleural HMGB1 on leukocyte recruitment, retention, lymphatic clearance, and ultimately, their impact on malignant disease.

The ability of adaptive cellular immunity to account for the myriad of pathogenic and endogenous threats is afforded through the diversity of the T cell and B cell receptors, collectively referred to as the adaptome (28). Using PCR-multiplexed amplification and next-generation sequencing of TCR sequences from bulk T cell populations isolated from MPEs, we have found that high intrapleural HMGB1 levels resulted in substantial reduction in TCRδ and TCRγ diversity specifically. Such a decrease in breadth of diversity is likely accompanied by the expansion of one or several dominant clones that have been actively recruited to the MPE space (e.g., clonality). These findings suggest that HMGB1 release may be associated with induction of an antigen-specific γδ T cell response, or alternatively, that aberrant release of DAMPs liberates yet unidentified molecular antigens driving the recruitment of specific γδ T cell populations. The early recruitment of such cells to tissues such as the skin, lung, and gut is in part for them to regulate lymphatic fluid flux and clearance of DAMPs, pathogens and cancer (39–43). Notably, decreased T cell diversity, primarily within the αβ T cell population, has been identified in the setting of cancer (44–46). Furthermore, TCRβ diversity has been associated with better patient responses to immunotherapy during checkpoint treatment for lung cancer (47). Herein, we have observed that cultured healthy donor γδ T cell proliferation was inhibited by rHMGB1, enhanced in the presence of acellular MPE fluid, and further augmented with addition of neutralizing HMGB1 antibody. Given the limited inflammatory cell migration in the setting of MPE, enhanced intrapleural HMGB1 concentrations could inhibit (1) infiltration of circulating γγδ T cells and (2) subsequent proliferation of all but the most reactive clonotypes, limiting repertoire diversity. Alternatively, recognition of non-peptide stress antigens (MICA/B; ULBP1-6) in the pleural environment could result in clonal expansion and reduction in repertoire diversity (48). Increased understanding of the biologic role of TCR diversity in health and disease has broad implications for cellular immunity and identification of specific and effective clonotypes, potentially useful for adoptive cell transfer therapy.

The effects of HMGB1 on T cell proliferation and phenotype are dependent on the source of HMGB1, resulting from tumor or myeloid cells, and the T cell activation status (35). Secreted HMGB1 from activated DCs results in CD4 Th1 polarization and expansion that is limited in the presence of a HMGB1 blocking antibody. HMGB1 mediated clonal expansion was dependent on CD3 and CD28 crosslinking and T cells are more sensitive to HMGB1 secreted by mature DCs than recombinant HMGB1 (49). rHMGB1 enhances CD4 proliferation at suboptimal doses of plate-bound OKT-3, but has limited effects on CD8 T cells (50). rHMGB1 induces CD4 Th17 polarization and apoptosis of regulatory cells with diminished IL-10 production (51). Although our findings suggest that increased HMGB1 levels within MPEs are capable of promoting T cell recruitment, further studies are necessary to determine the phenotype of recently recruited lymphocytes and if such cells are endowed with tumor-specific reactivity.

γδ T cells are MHC unrestricted effector cells that recognize non-peptide antigens, with an underappreciated role in tumor immune surveillance. Similar to their αβ T cell cousins, γδ T cells are highly susceptible to the composition of the tumor microenvironment, which may impart either antitumor or immunoregulatory capabilities depending upon the local signaling context (52). γδ T cells have been previously identified in the MPE of patients with NSCLC. Compared to circulating patient lymphocytes, γδ T cells in the MPE were found to have a predominant Vδ1/Vδ1-Vδ2- subtype and decreased expression of CD27 and CD28, with a suggested impaired activation and cytokine releasing state (53). In a murine model of Lewis lung carcinoma-derived MPE, IL-10 deficiency led to increased γδ T cell intrapleural proliferation and IL-17a production, reduced MPE volume, and longer survival that was dependent on γδ T cells. However, IL-10^–/–^ KO γδ T cells expressed lower levels of NKG2D and FasL, typically associated with activated γδ T cells (54, 55). Adding further complexity, γδ T cell-derived IL-17 mediates both anti-tumor and pro-tumor effects that are temporally regulated (56–58). Our findings suggests that in the presence of acellular MPE fluid γδ T cells are simultaneously driven to proliferate, while restricted in their ability to mount a cytokine response. Such processes may drive terminal exhaustion of this effector population leading to immune evasion and tumor escape.

## Data Availability Statement

The data will be available on request without restriction through the corresponding author.

## Ethics Statement

Studies have been approved by the Institutional Review Boards of the Shanghai Cancer Institute, the National University of Singapore, and the University of Pittsburgh. The patients/participants provided their written informed consent to participate in this study.

## Author Contributions

AS, AP, PM, RD, and ML wrote the main body of the text. KJ, AP, YW, PM, KR, MB-S, J-MY, SM, and AS assisted in data collection, data analysis, and figure preparation. SM performed cytologic analysis and figure preparation. AS, KJ, AP, PM, YW, RD, AL, J-MY, JH, and ML assisted in experimental design and manuscript review and editing. All authors contributed to the article and approved the submitted version.

## Conflict of Interest

MB-S and JH were employed by iRepertoire, Inc.

The remaining authors declare that the research was conducted in the absence of any commercial or financial relationships that could be construed as a potential conflict of interest.

## Supplementary Material

FIGURE S1 HMGB1 levels are elevated in MPEs secondary to lung cancer. HMGB1 levels were measured by specific ELISA in MPEs from lung (*N* = 11), breast (*N* = 19), and ovarian (*N* = 8) cancer patients. HMGB1 levels were significantly higher in lung cancer MPEs when compared to levels within ovarian MPEs (*p* = 0.025) and elevated in comparison to levels in breast cancer MPEs (*p* = 0.057). Data represent means ± SEM and Mann–Whitney U tests were used for comparisons.
