# Supplementary figures and images for: HMGB1 Promotes Myeloid Egress and Limits Lymphatic Clearance of Malignant Pleural Effusions
Source: Front Immunol. 2020 Sep 4;11:2027. doi: 10.3389/fimmu.2020.02027 (PMC7498625; doi:10.3389/fimmu.2020.02027)

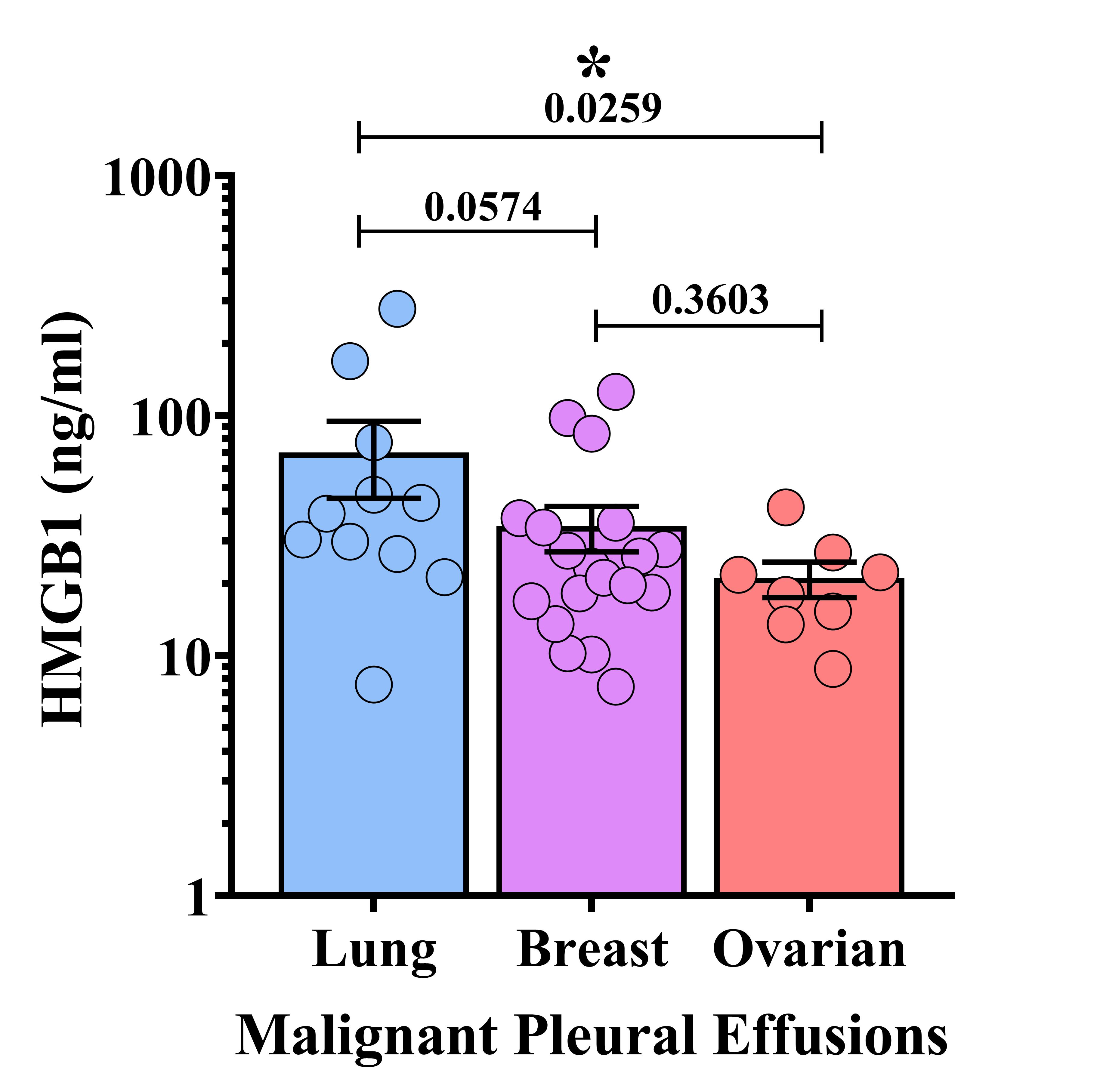

Supplement: FIGURE S1 — HMGB1 levels are elevated in MPEs secondary to lung cancer. HMGB1 levels were measured by specific ELISA in MPEs from lung (N = 11), breast (N = 19), and ovarian (N = 8) cancer patients. HMGB1 levels were significantly higher in lung cancer MPEs when compared to levels within ovarian MPEs (p = 0.025) and elevated in comparison to levels in breast cancer MPEs (p = 0.057). Data represent means ± SEM and Mann–Whitney U tests were used for comparisons. [file Image_1.jpeg]
